# The biochemistry of cell death: Cell Death: Apoptosis and Other Means to an End, Second Edition by Douglas R. Green, St. Jude Children’s Research Hospital, Cold Spring Harbor Laboratory Press, New York, 2018

**DOI:** 10.1038/s41419-020-2465-5

**Published:** 2020-04-20

**Authors:** Carlotta Zampieri, Carlo Ganini, Gerry Melino

**Affiliations:** Department of Experimental Medicine, TOR, University of Rome Tor Vergata, 00133 Rome, Italy

**Keywords:** Apoptosis, Autophagy

Can we impress you with a mind-blowing revelation? “Cells are not eternal in our bodies, but rather they encounter death!”^1^.

If someone pronounces this sentence at a biology class, students will laugh. Why? Because cell death has been studied since the 60s of the last century. When we speak about apoptosis nowadays, we regard it as a well-known truth. Anyway, this truth did not catch the attention of scientists until the 1980s, where the interest in the field exploded, leading to a dramatic increase of publications. Cell death passed “from neglect to hysteria” in only a few years, citing Martin Raff^2^, one of the founders of the field.

Undoubtedly, cell death is fundamental in our organism throughout the whole lifespan of a human being^3^: during body development, in tissue remodeling, and in many diseases like cancer, auto-immune, or infectious diseases. Many of these morbidities may originate from an incorrect apoptosis process: either from an excessive removal of essential cells for the body or from a defective elimination of potentially damaged cells. Our knowledge on apoptosis and cell death in general has reached an interesting point and we have a broad understanding of the mechanisms that are crucial to its regulation and of their consequences, making it reasonable to look at them in a therapy-oriented fashion.

Since we know so much about this field and the different forms of cell death^4^, does it make sense to write a book on cell death? Moreover, does it make sense to write a “second edition” to a previously published work? The answer is undoubtfully yes… and who better than Douglas Green could do it?

Douglas Green is one of the worldwide leading experts on apoptosis and cell death. He has dealt with the role of cell death in the regulation of cancer and immune response, studying the molecular events that drive the process, focusing on the induction-activation of apoptosis in T cells and the role of Myc, death receptors and Bcl-2 in this context. His deep understanding of the field allowed him to write the first edition of this book in a clear and straightforward manner, enriched by extremely informative figures. His talent as a biologist, as well as a communicator, has been condensed in this second edition, as you will discover by reading it from the beginning to the end, “in a few sittings”… and again, as in the previous edition, some “aha!” moments will not be missing!

**Fig. 1:**
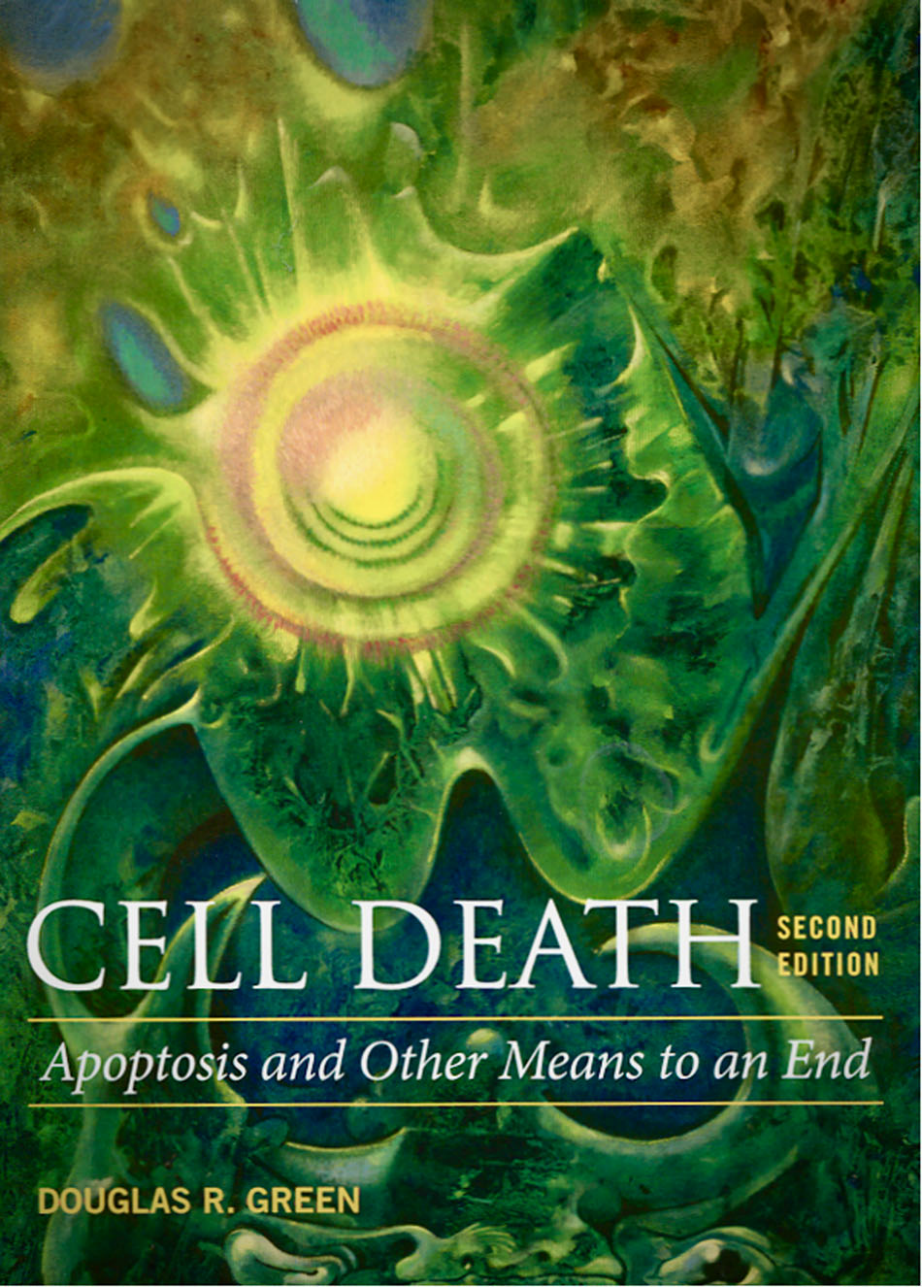
Cover of the second edition of the book “Cell Death: Apoptosis and Other Means to an End” by Douglas R. Green, edited by Cold Spring Harbor Laboratory Press, New York.

In this edition of “Cell Death: Apoptosis and Other Means to an End”^1^, the author extensively described the studies carried out on apoptosis so far, with outstanding clarity (Fig. 1). The theme has been addressed mostly under a biochemical point of view, starting from the description of the molecular mechanisms driving apoptosis, using an innovative bottom-up approach, as he pointed out, dissecting at first the extreme end of a cell’s life, tracing it back to the state of a healthy cell to discover what caused it to die. Nevertheless, this book might not be solely regarded as a comprehensive review about the biochemistry of the way cells die, since Douglas did not only focus on the molecular and cellular mechanisms driving cell death, but also described them with a glance at evolutionary biology, neuroscience, oncology, and immunology.

As a starting point, the author describes the caspases, the essential proteins responsible for the activation and the execution of apoptosis. From Douglas very words, we understand that the structure of the book is to be read from the beginning to the end, as each chapter builds up notions that will guide any reader to complete the picture presented in the following one, therefore allowing a personal reconnection of the dots, leading to the promised “aha!” moments. After the introduction on the caspases, the book moves on to the explanation of the main pathways responsible for their activation, the mitochondrial and the death receptors pathways. Anyway, cells do not only die by apoptosis: the other not apoptotic cell death types are briefly discussed alongside with the mechanisms by which the organism cleans the remaining from dead cells^4^. The various aspects of cell death described so far, are then used to summarize its role in specific contexts such as development and cancer.

The book ends with the future: the future of cell death!^5^ The author concludes with a formal approach applying mathematical models to discuss about the correctness, or incorrectness, of what we know on the topic and with an informal and more practical one, to understand how we could use our knowledge to engineer cell death. All the above is contained in a short didactic book with challenging topics, written in a pleasant key in less than 250 pages! A few details about each chapter will follow in the next paragraphs…

Chapter 1 is fundamentally an intriguing synopsis on cell death. If cell death’s increase can lead to cancer^6^, on the other hand its decrease can lead to infections or neurodegeneration. The organism must always maintain a balance between mitosis and apoptosis. In this chapter, the author outlines the molecular mechanisms that are regulating cell death: he explains the differences between passive death, occurring when the cell does not participate in the mechanism of death, and active death, when instead cells play an active role in the process.

Green’s bottom-up approach starts with the study of the biochemistry of apoptosis, which is elegantly presented in Chapters 2 and 3. Chapter 2 focuses on the key enzymes responsible for the activation and the execution of apoptosis, the caspases, through a chemical look (i) at their structure, (ii) at their domains (in diverse organisms), and (iii) at the structure of their substrates. Chapter 3 is more focused on the regulation of caspases activity by activators or inhibitors, grouping caspases into two types, depending on their function: initiator caspases (caspase-2, -8, -9, -10) or effector caspases (caspase-3, -6, -7). The power of these chapters relies on the linearity of the presentation of more than 30 years of the molecular characterization of apoptosis into a straightforward text, supported by highly informative figures… a good way to start learning about cell death (if you are facing it for the first time) or to make a point after a few years working on it!

Already at this point, the reader has a clear picture of how apoptosis occurs in a cell… but when is it activated? And how does the programmed cell death build up? Chapters 4 and 5 are there to answer our questions, focusing on the mitochondrial pathway of apoptosis, following the cytochrome c release by the mitochondrion, giving start to apoptosis in a process called mitochondrial outer membrane permeabilization (MOMP)^7^. MOMP, and consequently apoptosis, is closely regulated by a family of proteins, Bcl-2 whose importance is highlighted in Chapter 5 and fully described in its ability to mediate various signals inducing cell death^7^.

Anyway, the mitochondrial pathway is not a unique way to trigger apoptosis: a full chapter is therefore dedicated to the death receptors, which are activated by the death ligands. Death ligands are part of a family of proteins, the TNF (tumor necrosis factor) family, and death receptors are part of the tumor necrosis factor receptors (TNFRs) family. The chapter does not only focus on the description at a structural level of this alternative apoptosis triggering pathway, but also shows contacts between the activation of the death receptors and the mitochondrial pathway of apoptosis. A quick look at other modalities of apoptosis engagement is given in Chapter 7, where the inflammasome and other caspases activation platforms are described, remanding us about the close link between apoptosis and immunity, so dear to Douglas Green throughout his entire career.

Nevertheless, apoptosis is not the only way for a cell to die^4^. Therefore, in this comprehensive book on cell death, Green dedicates an entire chapter to describe the various types of cell death (necrosis, necroptosis, autophagy). Indeed, apoptosis and necrosis were the first cell death types studied. Necrosis is presented as an accidental death, caused by cell damage, although we now know that it can occur in a regulated fashion, called necroptosis, which is briefly discussed. Along with necrosis and necroptosis, the chapter also overviews the survival mechanism of autophagy.

The book, in addition to the biochemical side, includes the evolutionary aspects of cell death. It presents, as Green says, a “Just So Story”, a scenario of how the mitochondrial pathway of apoptosis could have evolved during eukaryotic evolution.

At this point a question in the readers’ brain might arise, one of those questions human beings have always asked themselves: what does happen after (cell) death? In Chapter 9, Douglas talks about “the burial, the disposing of the corpse” briefly focusing on phagocytosis which prevents the inflammatory response and cleans the tissues. The cell which is about to die releases “find-me” signals to be recognized. On the surface of the apoptotic cell the “don’t eat me” signals are blocked, and instead “eat-me” signals are activated by the dead cell-phagocyte interaction, leading to cell incorporation and degradation, not without any “consequences”.

Now the reader has a broad perspective on cell death and apoptosis. Therefore, it is about time to describe cell death regulation either during embryonic development, where apoptosis is also implicated in neuronal and lymphocytes selection. This topic is covered in Chapter 10, where the signals engaging cell death pathways are described in their role in the sculpting embryo.

Finally, talking about defective cell death, a chapter needs to talk about cell death and cancer, of course^6^. Much of the focus is on p53 and on its key role in apoptosis^8^; the author describes how p53 regulates apoptosis and inserts a clear and schematic panel on the protein regulation. But most of all, this chapter explains how “damaged” apoptosis can lead to cancer and how this mechanism might be exploited for cancer prevention. Once again, Douglas gives us a didactical and rational explanation of how cancer might be considered a disease caused by defective cell death.

The true ending of the book is outstanding. After a great deal of information in the previous chapters, the reader now understands how a cell engages cell death pathways, their regulation, how death occurs and how it is processed. But is the “big picture” we are looking at, really complete? To answer this question, the first part of the final chapter applies a mathematical approach to understand if the models we generate conform to our observations. But the true conclusion is a practical one: how can we use the information we have to engineer cell death? You just have to read the book, and you will also get to this point!

The book’s strength relies on its linearity, with a clear and consequential division of the chapters. It deals with complex and vast topics, but it remains easy-to-read and pleasant. It is a good reading for those who need a general overview of cell death. Douglas Green has in fact managed to schematize the engaged processes to the right point and in less than 250 pages, without being repetitive and without making it as a boring list of reactions.

The molecular interactions are well schematized in the figures—even if sometimes with synthetic legends—through stylish and plain images, not too colorful. Whilst also beginners are able to read this book, it is suitable for a diverse audience (without neglecting the rich “additional reading” section, full of keystone papers on cell death). Why should we read “Cell Death: Apoptosis and Other Means to an End”? Because reading this book: “cannot tell us whether we are right or wrong but, instead, where our ignorance lies and where our knowledge is useful”^1^.
